# Long non‐coding NR2F1‐AS1 is associated with tumor recurrence in estrogen receptor‐positive breast cancers

**DOI:** 10.1002/1878-0261.12704

**Published:** 2020-06-08

**Authors:** Anna Sanchez Calle, Tomofumi Yamamoto, Yumi Kawamura, Ai Hironaka‐Mitsuhashi, Makiko Ono, Hitoshi Tsuda, Akihiko Shimomura, Kenji Tamura, Fumitaka Takeshita, Takahiro Ochiya, Yusuke Yamamoto

**Affiliations:** ^1^ Division of Cellular Signaling National Cancer Center Research Institute Tokyo Japan; ^2^ Ph.D. Program in Human Biology, School of Integrative and Global Majors University of Tsukuba Ibaraki Japan; ^3^ Department of Medical Oncology Cancer Institute Hospital Tokyo Japan; ^4^ Department of Basic Pathology National Defense Medical College Saitama Japan; ^5^ Department of Pathology National Cancer Center Hospital Tokyo Japan; ^6^ Department of Breast and Medical Oncology National Cancer Center Hospital Tokyo Japan; ^7^ Department of Functional analysis National Cancer Center Research Institute Tokyo Japan; ^8^ Institute of Medical Science Tokyo Medical University Tokyo Japan

**Keywords:** dormancy, ER‐positive breast cancer, late recurrence, lncRNA, PR/ER transcriptional complex

## Abstract

The tenacity of late recurrence of estrogen receptor (ER)‐positive breast cancer remains a major clinical issue to overcome. The administration of endocrine therapies within the first 5 years substantially minimizes the risk of relapse; however, some tumors reappear 10–20 years after the initial diagnosis. Accumulating evidence has strengthened the notion that long noncoding RNAs (lncRNAs) are associated with cancer in various respects. Because lncRNAs may display high tissue/cell specificity, we hypothesized this might provide new insights to tumor recurrence. By comparing transcriptome profiles of 24 clinical primary tumors obtained from patients who developed distant metastases and patients with no signs of recurrence, we identified lncRNA NR2F1‐AS1 whose expression was associated with tumor recurrence. We revealed the relationship between NR2F1‐AS1 and the hormone receptor expressions in ER‐positive breast cancer cells. Gain of function of NR2F1‐AS1 steered cancer cells into quiescence‐like state by the upregulation of dormancy inducers and pluripotency markers, and activates representative events of the metastatic cascade. Our findings implicated NR2F1‐AS1 in the dynamics of tumor recurrence in ER‐positive breast cancers and introduce a new biomarker that holds a therapeutic potential, providing favorable prospects to be translated into the clinical field.

## Graphical abstract

We identified the long noncoding RNA NR2F1‐AS1 in the dynamics of tumor recurrence in ER‐positive breast cancers. NR2F1‐AS1 steers cancer cells into quiescence‐like state by the upregulation of dormancy inducers and pluripotency markers, and activates the metastatic cascade. Its expression is mediated by the PR/ER transcriptional complex bringing favorable prospects for developing new therapeutic strategies and introducing a new biomarker.

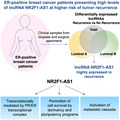

**Abbreviations**
bHLH: basic helix loop helix
BMP: bone morphogenic protein
ChIP: chromatin immunoprecipitation
DTC: disseminated tumor cell
EMT: epithelial‐to‐mesenchymal transition
ER: estrogen receptor
lncRNA: long noncoding RNA
NR2F1: nuclear receptor subfamily 2, group F, member 1
NR2F1‐AS1: NR2F1 antisense RNA 1
PR: progesterone receptor

## 1 Introduction

Nearly 10–40% of women with estrogen receptor (ER)‐positive tumors develop metastases long after the cessation of their treatment, and metastasis is responsible for the majority of breast cancer deaths. The administration of therapy within the first 5 years substantially reduces the risk of local and distant recurrence; however, tumors may reoccur 10–20 years after initial diagnosis (Pan *et al*, 2017; Zhang *et al*, 2013). Time‐to‐recurrence varies between tumor types. ER‐negative tumors are more aggressive, and the relapse tends to reoccur at around 2–5 years after diagnosis. In contrast, ER‐positive tumors have lower risk of recurrence in the first 5 years after diagnosis (Early Breast Cancer Trialists’ Collaborative Group (EBCTCG), 2005; Hess *et al*, 2003). Hence, metastases in ER‐positive subtypes generally become clinically apparent after long asymptomatic periods. It has been suggested that the variability in time‐to‐recurrence may be related to the ability of specific cancer cells to disseminate, colonize distant tissues, and establish premetastatic niches (Gomis and Gawrzak, 2017; Zhang *et al*, 2013). Disseminated tumor cells (DTCs) enter dormancy in secondary organs and remain dormant for extended periods (Aguirre‐Ghiso and Sosa, 2018; Gomis and Gawrzak, 2017). Thus, late recurrence is thought to arise from awaken proliferative DTCs. Decisive factors in the dynamics of the dormant‐to‐awaken switch seem to reside in the microenvironment. Factors such as TGFβ2, BMPs, GAS6, NR2F1, and DEC2, which are involved in the regulation of stem cell fate and pluripotency, are in fact dormancy inducers (Aguirre‐Ghiso *et al*, 2013; Bragado *et al*, 2013; Sosa *et al*, 2014; Sosa *et al*, 2015). Although the development of new models recapitulating dormancy programs has provided great insights into metastatic processes, the mechanisms that steer DTCs into quiescence are yet unclarified (Aguirre‐Ghiso and Sosa, 2018; Sosa *et al*, 2014).

Long noncoding RNAs (lncRNAs) are transcripts with more than 200 nucleotides, generally expressed at low levels, which can display high tissue/cell‐specific activities, and are involved in multiple mechanistic roles of gene and genome regulation (Sanchez Calle *et al*, 2018; Ulitsky & Bartel 2013; Li *et al*, 2015). Because of their involvement in disease and developmental defects, lncRNAs have gained more attention as possible biomarkers or therapeutic targets. In the context of cancer, increasing evidence supports the implication of lncRNAs in tumor suppression and tumorigenesis. Specifically, in breast cancer, several lncRNAs have been assigned cooperative functions in tumorigenesis (Tracy *et al*, 2018). The lncRNA MALAT1 was shown to contribute to tumor progression in ER‐positive (also known as luminal) cell lines and has been shown to control the expression of CD133 in the dedifferentiation process of breast cancer cells (Jadaliha *et al*, 2016; Zhang *et al*, 2012). Interestingly, the depletion of Malat1 in a metastasis‐prone transgenic mouse model of breast cancer reduced lung metastases; however, primary tumors were not different in size (Arun *et al*, 2016). HOTAIR has been proposed as predictive marker for metastatic progression and overall survival in early‐stage tumors of breast cancer (Gupta *et al*, 2010). On the other hand, NEAT1 and PTENP1, commonly known as tumor suppressors in various cancer types, have been shown to potentiate cell growth and tumor progression in breast cancer (Ke *et al*, 2016; Yndestad *et al*, 2017; Yndestad *et al*, 2018). Collectively, these nuances emphasize the tissue/cell specificity of lncRNAs and their ability to selectively target genes and impact signaling cascades in a confined manner.

We hypothesized that specific lncRNAs might be associated with late recurrence in breast cancer. To this end, we compared transcriptional profiles of primary tumors obtained from 10 recurrent and 14 nonrecurrent ER‐positive breast cancer patients. We successfully identified the lncRNA NR2F1 antisense RNA1 (NR2F1‐AS1) as a main lncRNA linked to recurrence. We unveil that the regulation of NR2F1‐AS1 expression is mediated by the transcriptional complex formed by progesterone receptor (PR) and ER, and show that its gain of function steers cancer cells into quiescence‐like state by the upregulation of dormancy inducers and the pluripotency, in addition to the activation of metastatic events. Thus, ER‐positive breast cancer cells expressing NR2F1‐AS1 could benefit of the activation of prosurvival signaling cascades, upregulate metastatic‐related biological processes, and bear the ability to enter dormancy.

## 2 Materials and Methods

### 2.1 Clinical specimens

Clinical specimens from luminal breast cancer patients were provided by the National Cancer Center Hospital (Tsukiji, Tokyo, Japan). This study was approved by the Internal Review Board of the National Cancer Center, Tokyo, Japan (no. 2013‐173) and conducted according to the Declarations of Helsinki, and all participants gave their written consent. In total, 24 primary tumor samples were collected with needle biopsy. Fourteen samples are considered as no recurrence (at least 10 years no recurrence observed) and 10 samples are the primary tumors from recurred patients within 10 years. Clinical information is shown in Table 1 and Table S1 (treatment information after surgery).

**Table 1:** Clinicopathological characteristics of luminal type breast cancer patients in this study

	Total (*n* = 24)	Number of patients (%)			*P* value
		Nonrecurrence (*n* = 14)	Recurrence (*n* = 10)		
Subtype					
Luminal A	14 (58)	10 (71)	4 (40)		0.123
Luminal B	10 (42)	4 (29)	6 (60)		
Invasive component (mm)					
<25	18 (75)	12 (86)	6 (60)		0.151
>25	6 (25)	2 (14)	4 (40)		
Histology					
IDC	23 (96)	14 (100)	9 (90)		0.227
ILC	1 (4)	0 (0)	1 (10)		
Ki67 score					
1	8 (33)	6 (43)	2 (20)		0.180
2	7 (29)	4 (29)	3 (30)		
3	7 (29)	2 (14)	5 (50)		
4	2 (8)	2 (14)	0 (0)		
Chemotherapy					
Yes	15 (63)	7 (50)	8 (80)		0.134
No	9 (38)	7 (50)	2 (20)		
Endocrine therapy					
Yes	20 (83)	11 (79)	9 (90)		0.459
No	4 (17)	3 (21)	1 (10)		
Metastasis					
Yes	10 (42)	0 (0)	10 (100)		<0.001
No	14 (58)	14 (100)	0 (0)		
			SLN	2 (20)	
			Cervical LN	1 (10)	
			Lung	2 (20)	
			Liver	3 (30)	
			Bone	3 (30)	
			Others	1 (10)	

### 2.2 Cell lines, culture conditions, and transfections

The cell lines were purchased from ATCC in 2016 and authenticated using STR profiling. All cell lines were routinely cultured in RPMI 1640 supplemented with 10% FBS without antibiotics at 37 °C and 5% CO_2_. The plasmids transfected into the BT474 cell line were pcDNA3.1‐P2A‐eGFP containing the sequence of the short Var4 of NR2F1‐AS1 and pcDNA3.1/Hygro(+) containing the sequence of the long Var1 of NR2F1‐AS1; pcDNA3.1‐P2A‐eGFP and pcDNA3.1/Hygro(+) were used as negative controls (GenScript, Piscataway, NJ, USA). Transfection was performed by nucleofection using the same parameters (Nucleofector™ 2b Device, Lonza Bioscience, Basel, Switzerland).

### 2.3 RNAi

Cell lines were transfected for 72 h with 5 nm target siRNA or the negative control No. 1 Silencer® Select, Ambion #4457171 (Life Technologies, Tokyo, Japan) using Lipofectamine™ RNAiMAX #13778075 (Invitrogen, Thermo Fisher Scientific, Tokyo, Japan). The sequences targeting human ER and PR are described in Table S2.

### 2.4 RNA isolation and quantification

cDNA was synthesized from 1 μg of total RNA isolated from tissue or cells using the High‐Capacity cDNA Reverse Transcription Kit #4374967 (Applied Biosystems, Tokyo, Japan). Target genes were detected using probes from TaqMan Gene Expression Assays (Thermo Fisher Scientific, Tokyo, Japan) or using specific primers as shown in Table S2 with Platinum™ SYBR™ Green qPCR SuperMix‐UDG (Thermo Fisher Scientific). Threshold cycle values were normalized to ACTB, and relative expression levels of target genes were calculated using the delta CT method.

### 2.5 Chromatin immunoprecipitation assay

A commercially available SimpleChIP^®^ Plus Kit (Magnetic 150 Beads) #9005 (Cell Signaling Technology, Japan, K.K.) was used according to the manufacturer’s instructions. Antibodies to progesterone receptor (Cell Signaling Technology, 6A1, #3172) and estrogen receptor α (Cell Signaling Technology, Tokyo, Japan, D8H8, #8644) are used. The PCR primers for the promoter region of NR2F1‐AS1 are shown in Table S1.

### 2.6 Immunoblotting

Western blotting was performed with 15–20 μg of protein lysate and 4–20% Mini PROTEAN® TGX™ Precast Protein Gels #4561095 (Bio‐Rad Laboratories, PA, USA). Antibodies to Stat1 (Cell Signaling Technology, #9172), phospho‐Stat1 (Cell Signaling Technology, #9177), p38 MAPK (Cell Signaling Technology, D13E1, #8690), phospho‐p38 MAPK (Cell Signaling Technology, D3F9, #4511), p21 Waf1/Cip1 (Cell Signaling Technology, 12D1, #2947T), and p27 Kip1 (Cell Signaling Technology, D69C12, #3686T) were used. For others, the same antibodies were used as for the chromatin immunoprecipitation (ChIP) assay, with the addition of mouse mAb anti‐actin antibody, clone C4 #MAB1501 (Merck Millipore, Burlington, MA, USA).

### 2.7 Anoikis assay

For anoikis analysis, we used CytoSelect Anoikis Assay (CBA‐081, Cell Biolabs, Inc., San Diego, CA, USA). NR2F1‐AS1‐variant 1, variant 4, or control plasmid was transiently transfected with lipofectamine 3000 reagent (Invitrogen, Thermo Fisher Scientific). The transfected cells (each well: 4 × 10^4^ cells) were plated into normal and anchorage‐resistant 96 well plates. MTT assay and fluorometric assay with calcein‐AM (green fluorescence, live cells) and EthD‐1 (red fluorescence, dead cells) were performed following the manufacturer’s instructions. The rate of anoikis resistance was estimated by comparing cell viability and cell death rate between normal and anchorage‐resistant condition.

### 2.8 In vivo analysis

NR2F1‐AS1‐variant 1 and control plasmids were transiently transfected into BT474 cells with lipofectamine 3000 reagent (Invitrogen, Thermo Fisher Scientific). Two days after transfection, the transfected cells (5 × 10^5^ cells) were intravenously transplanted into immunodeficient mouse. Three days after injection, the mice were euthanized and dissected, and lung tissues were collected. Metastasized cells were detected by quantitative PCR of gDNA with human‐specific primer (Funakoshi *et al*., 2017) and mouse‐specific primer (Duleba *et al*., 2020) as shown in Table S2.

### 2.9 Microarray

Total RNA was amplified and labeled with Cy3 using a Low Input Quick Amp Labeling Kit, one color (Agilent Technologies, Tokyo, Japan), following the manufacturer’s instructions. For each hybridization, 0.60 μg of Cy3‐labeled cRNA was fragmented and hybridized at 65 °C for 17 h to an Agilent SurePrint G3 Human GE v2 8x60K Microarray (design ID: 039494). The microarray chips were scanned using an Agilent DNA microarray scanner. Intensity values for each scanned feature were quantified using agilent feature extraction software version 11.5.1.1, which performs background subtraction. Normalization was performed with agilent genespring version 13.1.1 (per chip: normalization to 75th percentile shift). The altered transcripts were quantified using the comparative method. Raw and normalized microarray data are available in the Gene Expression Omnibus database (accession numbers GSE128600 and GSE128617). The intensity values were log2‐transformed and imported into Partek Genomics Suite 6.6 (Partek Inc., Chesterfield, MO, USA). One‐way analysis of variance was performed to identify differentially expressed genes. Fold change and *P*‐values were calculated for each analysis. Unsupervised clustering and heat map generation were performed with sorted datasets by Pearson’s correlation or Ward’s method with selected probe sets by Partek Genomics Suite 6.6.

### 2.10 Dataset sources

The clinical TCGA datasets for breast cancer (TCGA‐BRCA) were downloaded from the data portal of the Genomic Data Commons (GDC, https://portal.gdc.cancer.gov/projects/TCGA‐BRCA). Kaplan–Meier plots of overall survival (OS) and distant metastasis‐free survival (DMFS) were estimated for breast cancer with the complete analysis tool KM plotter (www.kmplot.com). Gene set enrichment analysis (GSEA) and Ingenuity pathways analysis (IPA) were used. Activated upstream regulators were considered when the IPA activation z‐score value was between 2‐ and 4‐fold (*P* < 0.001). For IPA, the analysis was performed following the manufacturer’s instructions (https://www.qiagenbioinformatics.com/products/ingenuity‐pathway‐analysis/).

### 2.11 Statistics

Data are presented as mean ± SD of *n* = 3 biological samples in triplicate. For two group comparisons, the statistical significance was determined by Student’s t‐test or Chi‐square test. For multiple comparisons, the significance of differences in average values was analyzed using one‐way ANOVA with Tukey’s HSD or Dunnett’s post hoc test. The limit of statistical significance for all analyses was defined as *P < 0.01 and **P < 0.001. For analyses of TCGA_BRCA datasets, Kruskal–Wallis and Wilcoxon tests were applied when *P* < 0.05 by Shapiro–Wilk test.

## 3 Results

### 3.1 Transcriptome analysis of 24 ER‐positive breast primary tumors

**Fig. 1:**
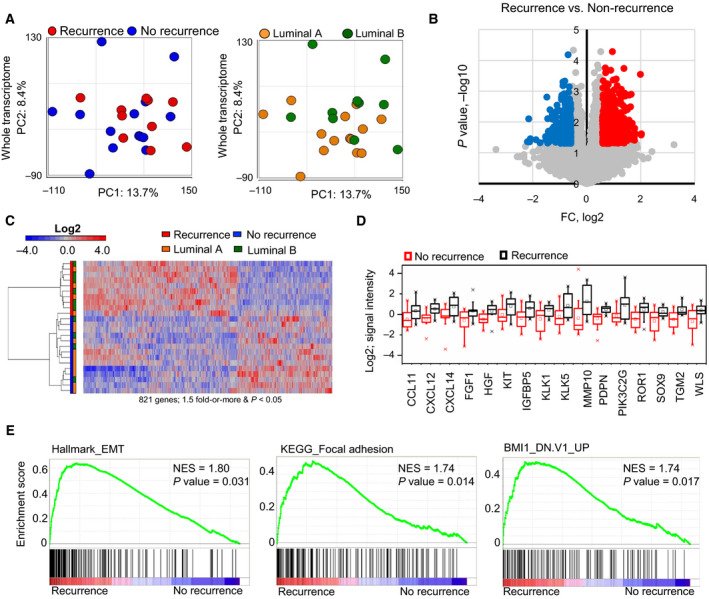
Molecular characterization of ER‐positive breast cancer primary tumors. (A) Principal component analyses (PCAs) of the whole transcriptome based on recurrence status (left) and luminal subtype (right) by microarray. (B) Volcano plot of the whole transcriptome data. The genes with 1.5‐fold or more change and *P* < 0.05 are marked with color (red: high and blue: low). (C) Heatmap based on the differentially expressed gene (DEG) probes clustering by recurrence status (1.5‐fold change, *P* < 0.05). (D) Selected DEGs upregulated in the recurrence group. Upper whisker: max, and lower whisker: min. (E) Representative gene set enrichment analysis (GSEA) for recurrence versus no recurrence. NES = normalized enrichment score (*P* < 0.05).

To elucidate a distinctive molecular signature of recurrence, we performed transcriptome analysis with nontreated 24 clinical needle‐biopsied samples from ER‐positive breast primary tumors (Table 1): 10 tumors which recurred after the treatment and 14 which did not recur. Principal component analysis (PCA) for the whole transcriptome did not show the clear separation among recurrence status as well as luminal subtypes (Fig. 1A). Based on recurrence status, differentially expressed genes (DEG, Fig. 1B) were clustered, which did not match with luminal subtypes (Fig. 1C); however, a gain of cancer‐related genes in the recurrence group was clearly observed (Fig. 1D). Additionally, gene set enrichment analysis (GSEA) identified enriched gene sets related to EMT, focal adhesions, and cancer stem cell‐associated markers (*P* < 0.05) (Fig. 1E). Thus, the transcriptome data of primary tumors that recurred after the treatment suggest distinct expression profiles from the primary tumors that did not recur after the treatment.

### 3.2 LncRNA NR2F1‐AS1 is associated with recurrence

**Fig. 2:**
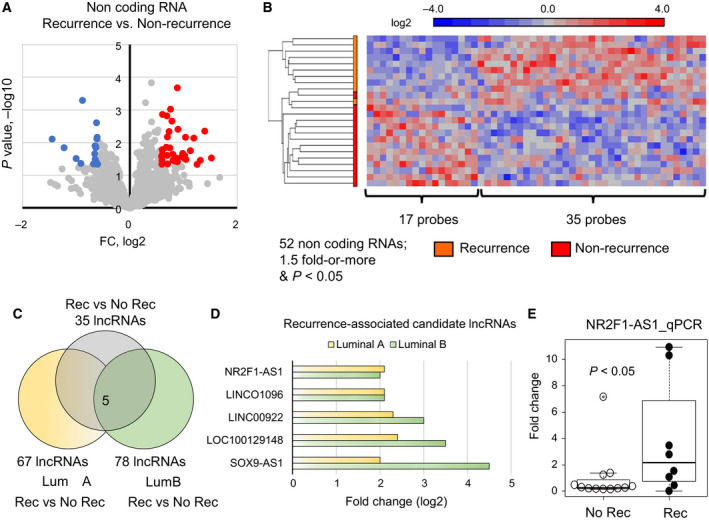
The NR2F1‐AS1 is associated with tumor recurrence. (A) Volcano plot of the lncRNA data. The genes with 1.5‐fold or more change and *P* < 0.05 are marked with color (red: high and blue: low). (B) Heatmap based on the differentially expressed lncRNA probes clustering by recurrence status (1.5‐fold change, *P* < 0.05). (C) Venn diagram showing that the lncRNA candidates with upregulated in the recurrence status. (D) 5 computationally predicted lncRNAs, which may be associated with tumor recurrence. (E) qPCR validation of NR2F1‐AS1 as the main lncRNA candidate associated with recurrence. Upper whisker: max, and lower whisker: min.

Since lncRNAs may display highly tissue/cell‐specific activities, we questioned whether this could represent an optimal feature to signify tumor recurrence in ER‐positive breast cancers. Thus, we analyzed the differentially expressed lncRNAs associated with tumor recurrence. When compared the expression of lncRNAs among recurrence status, only 35 lncRNAs were upregulated and 17 lncRNAs were downregulated in the tumors which recurred after the treatment (Fig. 2A). Although there were a few lncRNAs distinctly expressed, they enabled to separate the recurrence and nonrecurrence (Fig. 2B). To find out the lncRNAs which are associated with both luminal A and B types, we also compared separately and narrowed down to 5 candidates (Fig. 2C). The expressions of these lncRNAs were high in both luminal A and B types (Fig. 2D). Further validation by quantitative PCR confirmed NR2F1‐AS1 as a lncRNA that was likely related to recurrence (Fig. 2E). For other 4 candidates, we could see the trend showing higher expression in recurrent cases but not statistically significant (Fig. S1).

### 3.3 Clinical relevance of NR2F1‐AS1 in ER‐positive breast cancer

**Fig. 3:**
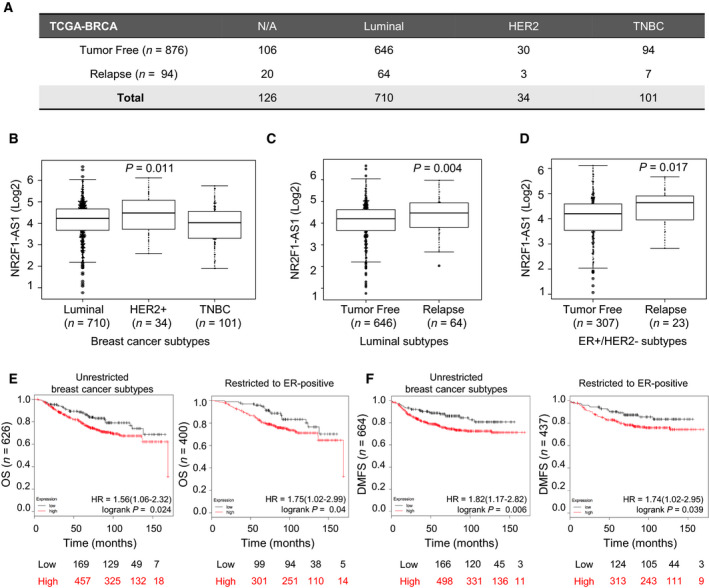
Biological relevance of NR2F1‐AS1 in breast cancer based on public database. (A) Summary table of clinical cases with track record of recurrence status. (B–D) Sequencing reads of TCGA_BRCA data for NR2F1‐AS1 in Luminal, HER2‐positive and TNBC subtypes (B), subtracted clinical cases with Luminal subtype (C), and ER‐positive/HER2‐negative subtype (D), Upper whisker: max, and lower whisker: min. (E, F) Kaplan–Meier plots for the overall survival (OS) (E) and distant metastasis‐free survival (DMFS) (F), unrestricted to breast cancer subtypes and restricted to ER‐positive cases (https://kmplot.com/analysis/). To separate samples, Auto select best cutoff was used.

To expand our knowledge about the presence of NR2F1‐AS1 in breast cancer subtypes, we analyzed datasets from The Cancer Genome Atlas Breast Cancer (TCGA_BRCA). Because of the differences in relapse between ER‐negative and ER‐positive subtypes, we stratified the datasets into 3 main phenotypes, luminal (ER+), HER2‐positive (ER−/PR−/HER2+), and TNBC (ER‐/PR‐/HER2‐), and extracted the cases with available information about relapse status (Fig. 3A). Interestingly, HER2‐positive subtypes display higher expression of NR2F1‐AS1 (*P* = 0.011, Fig 3B). However, when the cases with relapse we isolated, the presence of NR2F1‐AS1 was more prominent in ER‐positive luminal cases, although not statistically significant (*P* = 0.058, Fig. S2A). Thus, we subtracted the ER‐positive luminal subtypes and found a significant expression of NR2F1‐AS1 in recurrence group (*P* = 0.004, Fig 3C). Also, we found that the expression of NR2F1‐AS1 is significantly associated with the status of lymph node (Fig. S2B) and patients who received the initial diagnose under 50 years old (Fig. S2C).

On 2012, Curtis et al. introduced a novel classification of breast cancer subtypes based on the meta‐analysis of copy number variation from 2000 breast tumors. Recently, the same group has reported the associated risk of recurrence for each subtype (Curtis *et al*, 2012; Rueda *et al*, 2019). The latter study shows that the IntClust subtypes belonging to late recurring with highest risk of relapse up to 20 years are enriched in ER+/HER2‐. In line with these findings, we subtracted the ER+/HER2‐ cases and divided them accordingly to the relapse status. Strikingly, the relation between recurrence, ER+/HER2‐, and the expression of NR2F1‐AS1 was found significant (*P* = 0.017), supporting the association of NR2F1‐AS1 to late recurrence (Fig 3D). Additionally, using another public database, a Kaplan–Meier analysis of breast cancer patients indicated that high NR2F1‐AS1 levels correlated with poor overall survival (OS) and distant metastasis‐free survival (DMFS), even when restricted to ER‐positive cases (Fig 3E‐F).

### 3.4 ER and PR negatively regulate NR2F1‐AS1 transcription

**Fig. 4:**
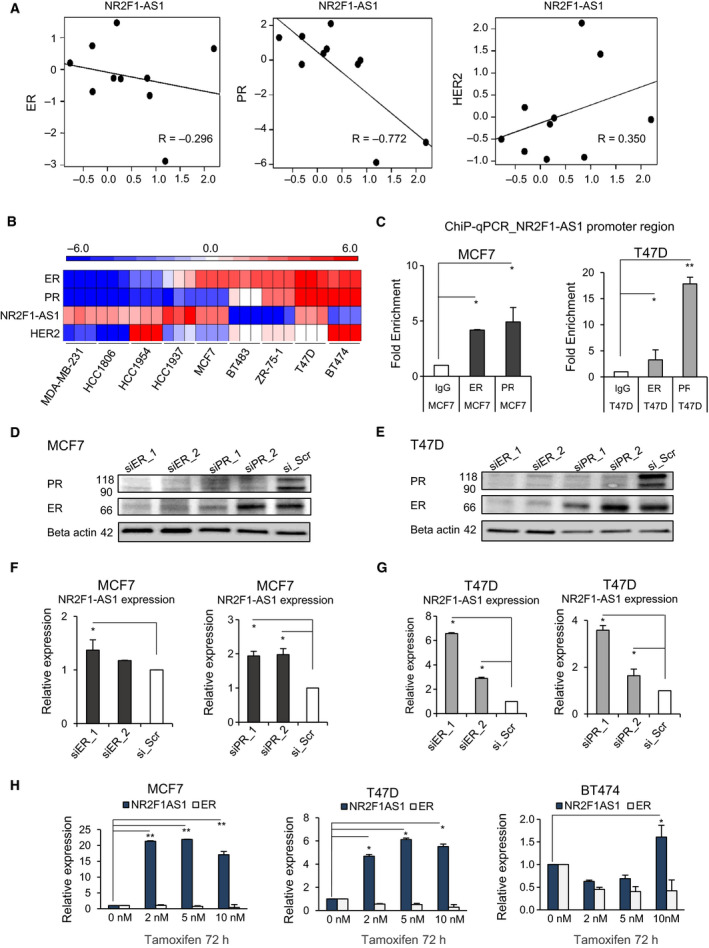
Progesterone receptor and ER govern NR2F1‐AS1 expression. (A) Pearson correlation of ER, PR, and HER2 with NR2F1‐AS1 restricted to recurrence samples based on microarray samples. (B) Heatmap showing the expression levels of ER, PR, NR2F1‐AS1, and HER2 in a series of breast cancer cell lines of different origin based on qPCR. (C) ChIP‐qPCR for the enrichment of ER and PR in the promoter region of NR2F1‐AS1. Normal rabbit IgG is shown as a negative control. (D, E) Transient silencing of ER and PR for 72 h with siRNA in MCF7 (D) and T47D (E). Two different siRNA sequences were used for each receptor. Scramble siRNA (siR_Scr) is shown as a negative control. (F, G) Relative expression levels of NR2F1‐AS1 upon transient knockdown of ER and PR. (H) Relative expression levels of NR2F1‐AS1 and ER after the administration of low doses of tamoxifen for 72 h (*n* = 3 in triplicate; Student’s t‐test, *P < 0.01, **P < 0.001). Error bars: mean ± SD.

To understand whether NR2F1‐AS1 is related to the ER‐positive subtype, we first addressed whether its presence was associated with the hormone receptors. When only using recurrence cases, we noted an inverse correlation between NR2F1‐AS1 and PR (Fig 4A), although ER showed a weak correlation with NR2F1‐AS1. In contrast, no recurrence cases did not show a significant correlation with any hormone receptors (Fig. S3), suggesting that the presence of NR2F1‐AS1 is more tightly related to recurrence than ER‐positive subtype itself.

To interrogate the biological relevance of the presence of NR2F1‐AS1, we screened its expression in 9 genotypically distinct breast cancer cell lines. Briefly, we observed that NR2F1‐AS1 expression was higher in the absence of hormone receptors (Fig. 4B) and it is also confirmed by the correlation of NR2F1‐AS1 and hormone receptor expression in the cell lines (Fig. S4). Cell lines expressing higher NR2F1‐AS1 levels included those representatives of TNBC and HER2‐positive subtypes and, interestingly, the ER‐positive luminal type MCF7 and T47D cell lines. Other ER‐positive cell lines, such as BT483, ZR‐75‐1, and BT474 cells, showed no quantitative expression of NR2F1‐AS1. Notably, MCF7 and T47D lines are derived from metastatic sites of pleural effusion, while the rest of ER‐positive cell lines were originally derived from nonmetastatic sites. This finding prompted us to consider that the expression NR2F1‐AS1 is linked to the kinetics of metastasis.

Previous studies have reported that the physical interaction of PR and the ER transcriptional complex can activate and redirect transcriptional outputs in breast cancer cells (Carroll *et al*, 2017). Since our clinical recurrence samples showed an inverse correlation between PR and NR2F1‐AS1, we evaluated the potential chromatin binding of PR and ER to the NR2F1‐AS1 promoter region. We employed ChIP‐qPCR in the ER‐positive cell lines expressing higher levels of NR2F1‐AS1, namely MCF7 and T47D cells. A gain of enrichment for PR over ER was observed and was more apparent in T47D cells, which have markedly higher PR levels than MCF7 cells (Fig. 4C). Our data suggested that the transcriptional regulation of NR2F1‐AS1 is most likely mediated by PR. However, the inverse correlation was indicative of repression of NR2F1‐AS1 expression. To confirm this, we transiently knocked down the expression of PR and ER by siRNA (Fig 4D and E). Consistently, the expression levels of NR2F1‐AS1 increased upon the transient depletion of ER and PR in both cell lines (Fig. 4F and G).

To further confirm whether the ER‐PR signaling inhibits the expression of NR2F1‐AS1, we exposed 3 different ER‐positive breast cancer cell lines to low doses of tamoxifen for 72 h to avoid compromising cell viability. After the treatment of low doses of tamoxifen, the expression of ER decreased slightly, and the levels of NR2F1‐AS1 markedly increased in MCF7 and T47D (Fig. 4G). The BT474 cell line, which does not show detectable levels of NR2F1‐AS1, showed a slight increase in NR2F1‐AS1 expression when exposed to 10 nM of TAM for 72 h. Collectively, our data indicated that the ER–PR transcriptional complex negatively mediated the transcriptional expression of NR2F1‐AS1. Interestingly, in the early stage of ER‐positive breast cancer, high levels of PR are linked to decreased metastasis (Mohammed *et al*, 2015; Thomas and Gustafsson, 2015). Thus, we wondered whether this could relate to the presence of NR2F1‐AS1.

### 3.5 ER‐positive breast cancer cells undergo quiescence‐like state upon NR2F1‐AS1 overactivation

**Fig. 5:**
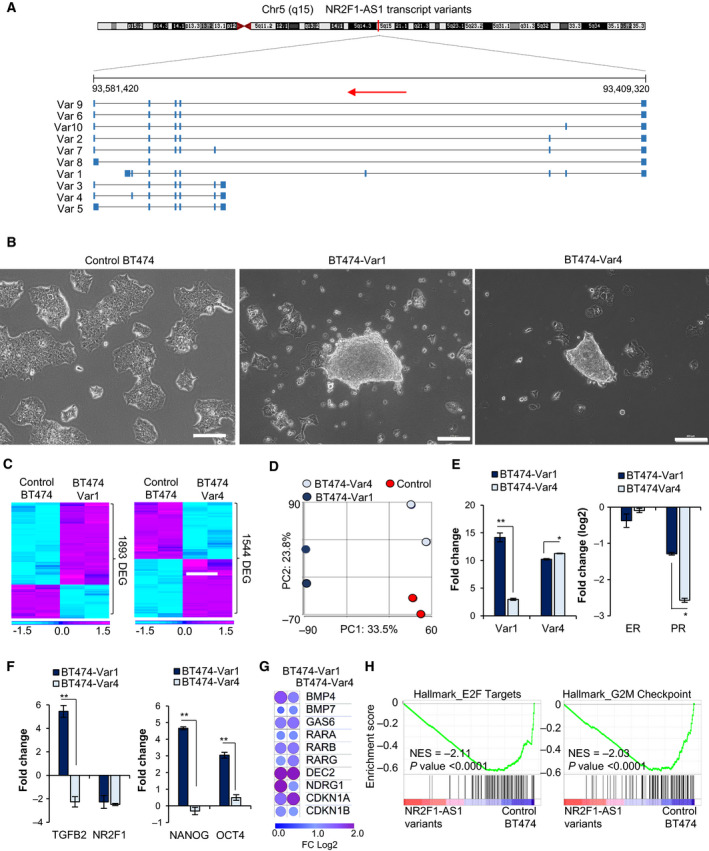
Gain of function of NR2F1‐AS1 Var1 and Var4 induce dormancy. (A) Illustration of the genomic sequence of Homo sapiens NR2F1‐AS1 and its transcript variants (indicated as Var). NC_000005.10 chromosome 5q15 GRCh38.p12. Source: NCBI Gene. (B) Morphological changes induced by the overactivation of NR2F1‐AS1 Var1 and Var4 in BT474 cells. Transfected variant and control BT474 cells underwent antibiotic selection for 20 days and were maintained in antibiotic‐free medium thereafter. Scale bars: 200 μm. (C) Heatmap based on DEG in Control BT474 cells versus BT474‐Var1 and BT474‐Var4 cells (*n* = 2 replicates, 2‐fold increase, *P* < 0.05). (D) PCA of the whole transcriptomes of Control BT474, BT474‐Var1, and BT474‐Var4 cells. (E, F) qPCR analysis of the fold change in expression levels (*n* = 3 in triplicate; Student’s *t*‐test, *P < 0.01, **P < 0.001). Fold changes are shown by comparing with control cells. Error bars: mean ± SD. (G) Heatmap based on dormancy related factors found in BT474‐Var1 and BT474‐Var4 cells, shown as log2 fold change. (H) Functional enrichment analysis by GSEA for BT474‐Var1 and BT474‐Var4 cells (in combination) versus Control BT474 cells (*P* < 0.05).

NR2F1‐AS1 is well conserved across species and is fundamentally involved in the transcriptional regulation of neuronal fate (Ang *et al*, 2019). It has known 10 different variants and is located on chromosome 5q15 (Fig. 5A). Prior to characterizing the outcomes of overexpressing NR2F1‐AS1, we confirmed that at least NR2F1‐AS1 variant 1 (Var1) and variant 4 (Var4) were present in the recurrence group of clinical samples. To test their function, we separately stably transfected them into BT474 cells, which originated from a primary tumor site and do not express NR2F1‐AS1. The overexpression of both variants attenuated cell proliferation and slowed cell growth. The decreases in percentage of Ki67‐positive cells were observed in BT474 cells with transient expression of NR2F1‐AS1 Var1 and Var4 (Fig. S5). Cell viability became progressively compromised, and cell population had dramatically reduced to a few cells. The remaining cells were maintained, and at 60 days, small colonies could be observed. After 75 days, colonies displayed remarkable morphological changes compared with control BT474 cells (Fig. 5B). Also, we confirmed p21 and p27 gene expression and protein levels in BT474‐Var1 and BT474‐Var4, and the increases in p21 and p27 levels were observed (Fig. S6).

With overexpression of both NR2F1‐AS1 variants, a large number of genes were differentially expressed (Fig. 5C). PCA mapping with whole transcriptome revealed that Var1 and Var4 showed distinct expression profiles (Fig. 5D). We confirmed that surviving colonies overexpressed their corresponding transfected NR2F1‐AS1 variants (Fig. 5E, left). Strikingly, the overexpression of Var1 induced the upregulation of endogenous Var4, but the converse was not observed, suggesting coordinated transcriptional activity. In line with our previous correlations, PR and ER were downregulated upon the overexpression of both NR2F1‐AS1 variants; in particular, the presence of Var4 seemed to exert a major effect on ER and PR expression (Fig. 5E, right).

Tumor cell dormancy can be fueled through distinct cues, such as the protein‐coding genes, TGFβ2 and NF2F1 (Bragado *et al*, 2013; Sosa *et al*, 2015). Thus, since BT474‐Var1 and BT474‐Var4 showed attenuated cell growth and proliferation, we assessed the expression of TGFβ2 and NR2F1 (Fig. 5F, left). Contrary to our expectations, only BT474‐Var1 cells displayed increased levels of TGFβ2, while NR2F1 was downregulated in both populations. Because quiescence status is closely related to the stemness for the survival of dormant cells (Aguirre‐Ghiso and Sosa, 2018), we also evaluated the pluripotent markers NANOG and OCT4, which were upregulated only in BT474‐Var1 cells (Fig. 5F, right). This finding underlined the functional divergence due to the simultaneous coexpression of the two NR2F1‐AS1 variants versus Var4 alone. Then, we examined the presence of commonly known dormancy inducers and cyclins involved in cell cycle arrest (Fig. 5G). Although there is diversity in their expression levels between Var1 and Var4, both had equal stimulation of the transcription factor differentially expressed in chondrocytes 2 (DEC2), which is known to induce dormancy (Aguirre‐Ghiso *et al*, 2013; Aguirre‐Ghiso and Sosa, 2018; Gomis and Gawrzak, 2017; Sosa *et al*, 2014). Next, we addressed the differentially represented pathways by GSEA and found that both populations strongly downregulated proliferation‐related pathways such as E2F targets and G2M checkpoints (Fig. 5H), as well as MYC targets and mitotic‐related processes (Fig. S7A). To further investigate the function of NR2F1‐AS1, we knocked down NR2F1‐AS1 by siRNA in MCF7 cell line (Fig. S8A). Although the knockdown of NR2F1‐AS1 was confirmed by qRT‐PCR, there was no significant change observed in the NR2F1‐AS1 knockdown cells. Moreover, GSEA could solely report a slight downregulation of the TGFβ signaling pathway in at *P* < 0.01 (Fig. S8B).

### 3.6 NR2F1‐AS1 may endow metastatic potential to ER‐positive breast cancer cells

**Fig. 6:**
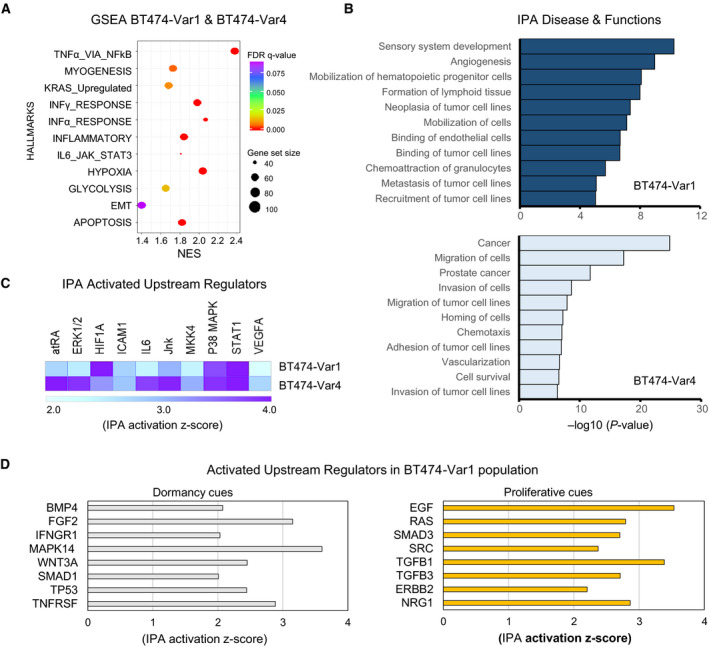
NR2F1‐AS1 promotes events of the metastatic cascade. (A) Functional enriched pathways in BT474‐Var1 and BT474‐Var4 cells by GSEA. Data are presented as FDR value, gene set size (50–100), and NES (normalized enrichment score). (B) Ingenuity pathway analysis (IPA) of biological functions and diseases. Selected biological functions are shown. Top, BT474‐Var1, and bottom, BT474‐Var4. (C) IPA for upstream regulators showing similarly activated regulators in BT474‐Var1 and BT474‐Var4 cells. (D) IPA of the activation of upstream regulators in BT474‐Var1 cells. Representing patterns of dormant (left) and proliferative (right).

We further scrutinized significantly enriched pathways in NR2F1‐AS1T474‐Var1 and BT474‐Var4 cells and found hypoxia and glycolysis, with predominant upregulation of immune‐related pathways based on GSEA (Fig. 6A). These representative pathways have been considered indicators of dormancy in previous studies with dormant hematopoietic stem cells (Cabezas‐Wallscheid *et al*, 2017) and nonproliferative cells from the inner mass of multicellular spheroids of colon carcinoma cells (Zhang *et al*, 2015). GSEA could only differentiate EMT and KRAS signaling enriched in BT474‐Var4 cells compared to BT474‐Var1 cells (Fig. S7B), suggesting that NR2F1‐AS1 variants mainly elicit the activation of similar pathways. Using ingenuity pathways analysis (IPA), we identified the top biological functions and diseases from the annotated genes that were differentially expressed in BT474‐Var1 and BT474‐Var4 cells (Fig. 6B). Notably, the overexpression of both variants increased biological functions encompassed in the metastatic network. Similar to GSEA results, BT474‐Var4 cells presented a remarkably enriched oncogenic signature compared to BT474‐Var1 cells. Hence, albeit both variants enhance the metastatic potential of BT474 cells, they may trigger a differential transcriptional response.

Next, we selected the upstream regulators that were commonly activated in BT474‐Var1 and BT474‐Var4 cells and were linked to dormancy programs (Fig. 6C). Among them, we found all‐trans retinoic acid (atRA), p38 MAPK, and STAT1. Importantly, atRA has been ascribed to sustain dormancy (Cabezas‐Wallscheid *et al*, 2017; Müller‐Hermelink *et al*, 2008). Similarly, proliferating squamous cell carcinoma cells entered dormancy and induced TGFβ2 in a p38‐dependent manner upon treatment with atRA (Sosa *et al*, 2015). Furthermore, high p38 MAPK and low ERK1/2 levels are required for tumor cell quiescence because the activation of p38 may induce growth arrest (Bragado *et al*, 2013; Sosa *et al*, 2014; Zhang *et al*, 2013). Another upstream regulator is STAT1, which has been implicated in the arrest of cell proliferation by means of JAK2/STAT1 (Vander Griend *et al*, 2005). The phosphorylation of STAT1 and p38 MAPK was examined (Fig. S9), and the result showed increased phosphorylation of STAT1 in both BT474‐Var1 and BT474‐Var4, although phosphorylation of p38 MAPK was observed only in BT474‐Var4.

Because the BT474‐Var1 population exhibited a higher degree of complexity, we unilaterally dissected its molecular signature. Among the activated upstream regulators, we could recognize patterns of dormancy and proliferative DTCs (Fig. 6D). Since late metastasis has been confined to the reactivation of dormant DTCs, these results highlighted the coexistence of cell subpopulations at different points of the dormant‐to‐awaken state. To resume proliferation, quiescent cells activate EGF, RAS, TGFβ1, and TGFβ3, which upregulation have been attributed to cause higher malignancy in breast tumors (Chen *et al*, 2015; Lo *et al*, 2006). To investigate the metastatic potential of NR2F1‐AS1, we firstly tested whether NR2F1‐AS1 influences anoikis resistance in BT474 cells. As shown in Fig. S10, both variants tended to increase anoikis resistance based on the cell viability and cell death in an anchorage‐independent condition. Next, we transplanted BT474‐Var1 cells intravenously into immunodeficient mice and examined the metastatic potential of NR2F1‐AS1 in the mouse lung, by detecting transplanted BT474 cells with qPCR of human specific gDNA primer. Although it was not statistically significant, BT474‐Var1 cells were more frequently detected than the control cells in the mouse lungs (Fig. S11). Collectively, our results suggest that NR2F1‐AS1 supports tumor cell survival by the activation of metastatic‐entailed events and dormancy programs, but it is not sufficient to sustain prolonged quiescence without the support of microenvironmental extrinsic factors.

## 4 Discussion

The metastatic cascade encompasses the events of invasion, neoangiogenesis, intravasation, dissemination, extravasation, dormancy, and colonization (Dasgupta *et al*, 2017; Giancotti, 2013). After tumor cells have extravasated in secondary organs, they may enter dormancy and remain dormant for long asymptomatic periods. Thus, late recurrences are thought to reoccur from awakened DTCs that establish premetastatic niches and colonize in the new tissue. We found that the expression of NR2F1‐AS1 variants activate biological processes relating to the metastatic cascade (Fig. 6B). Further enrichment of EMT, hypoxia, and inflammatory response pathways, along with activated upstream regulators such as HIF1α, VEGFA, and ICAM‐1, was also found in both populations (Fig. 6A,C). It is broadly accepted that circulating tumor cells must display an EMT signature to overcome hostile environments throughout the multistep metastatic cascade (Dasgupta *et al*, 2017). Chemokines that participate in the inflammatory response can regulate biological processes of cell differentiation and survival, and processes of neovascularization and extravasation require of the activation of VEGFA, hypoxia, and ICAM‐1 (Fig enschau *et al*, 2018; Nobre *et al*, 2018; Schröder *et al*, 2011). Recently, a report has shown the co‐regulation of hypoxia and dormancy programs in posthypoxic ER‐positive DTCs from patient‐derived xenografts (PDX) and a transgenic mouse model (Fluegen *et al*, 2017). Thus, it is likely that NR2F1‐AS1‐expressing tumor cells activate events of the metastatic cascade, including cell survival and dormancy.

The viability of NR2F1‐AS1‐transfected BT474 cells was seriously influenced (Fig. 5B). The activation of apoptotic signaling was confirmed by GSEA, alongside with prosurvival TNFα/NFκB signaling pathway that was strongly enriched (Fig. 6A). Interestingly, the dormancy inducer DEC2, which was found equally upregulated in BT474‐Var1 and BT474‐Var4, activates antiapoptotic signaling in breast cancer cells, and its expression appears to be regulated by TNFα/NFκB (Li *et al*, 2003; Olkkonen *et al*, 2015). Comparative analyses for the genomic occupancy sites, by chromatin isolation by RNA purification sequencing (ChIRP‐seq), suggested that NR2F1‐AS1 variants can bind to distinct genomic loci acting in trans, eliciting different transcriptional responses (Ang *et al*., 2019). The same study revealed that NR2F1‐AS1 has preference for binding DNA regions rich in basic helix loop helix (bHLH) motifs; bHLH proteins constitute a family of transcription factors implicated in circadian rhythm, cell differentiation, and hypoxia. Another bHLH family member DEC1 has been attributed to induce proapoptotic cues and mediate the repression of DEC2 (Li *et al*, 2003; Liu *et al*, 2010). Hence, the divergence in cell survival fate, dictated by the overexpression of NR2F1‐AS1, could hinge on the affinity of NR2F1‐AS1 variants for genomic regions enriched with bHLH motifs‐containing factors. This would impose a clonal selection on the cell population, whence residual cells expressing NR2F1‐AS1 would have activate the transcription of DEC2, steering tumor cells into quiescence.

In addition to slower cell cycle, the overactivation of NR2F1‐AS1 induced phenotypical changes in tumor cells that were apparent at transcriptomic level. Hierarchical clustering heatmap indicated 1893 DEG between BT474‐Var1 and control BT474, and 1544 DEG for BT474‐Var4 (Fig. 5C). Among these, PR and ER were found downregulated (Fig. 5D). These observations, together with the upregulation of NR2F1‐AS1 upon low doses of tamoxifen (Fig. 4H), prompted us to question whether NR2F1‐AS1 could serve as backup plan for the downregulation of ER and PR. This supposition became more consistent with preliminary drug screening on MCF7, in which residual cells displayed gradually enhanced NR2F1‐AS1 expression after the administration of combined treatment with TAM and the CDK4/6 inhibitor palbociclib (in a mol:mol ratio) for 5 days (Fig. S12). Therefore, ER‐positive breast cancer patients presenting high levels of NR2F1‐AS1 would be at an increased risk of recurrence when receiving endocrine therapies.

When IPA for the activated upstream regulators was restricted to BT474‐Var1, we observed 2 trends of molecular patterns corresponding to dormancy cues and proliferative cues (Fig. 6D; Sosa *et al*, 2014), indicating the existence of tumor cells at different points of the dormant‐to‐awaken state. The molecular intricacy of BT474‐Var1 should be given by the simultaneous expression of the two variants. Seemingly, the Var1 acts as main trigger of dormancy cues, with the activation of quiescence inducers and pluripotency markers, whereas, as indicated in the GSEA results (Fig. S2A, Fig. 6A), the activation of the Var4 would foster EMT and the upregulation of KRAS signaling, most likely supporting the resumption of proliferative cues. The activation of HER2/Neu signaling appears to be consequence of the overactivation of NR2F1‐AS1. Interestingly, DTCs are currently characterized by the expression of multi‐markers, and the positive expression of HER2 is commonly observed among DTCs of different cancer types (Harper *et al*, 2016; Hosseini *et al*, 2016).

Although the data presented here demonstrated that NR2F1‐AS1 expression is positively related to dormancy in luminal type breast cancer, one limitation of this study is that we could not identify the key molecules or signals of how the dormant cells wake and expand in the secondary tumors at distal organs. As shown in Fig. 6D, we found that BT474‐Var1 possessed both dormancy and proliferation cues, but the cell growth of BT474‐Var1 nearly stopped for the long term *in vitro*. One possible answer of how the cells wake up might be simply the silencing of NR2F1‐AS1 expression in breast cancer cells. To further investigate the dormant‐to‐awaken state in breast cancer, novel model *in vitro* and *in vivo* would be necessary to screen the key factors for waking up the cells from dormancy.

## 5 Conclusions

Collectively, we identified the biological relevance of NR2F1‐AS1 in the kinetics of tumor recurrence in ER‐positive breast cancers and elucidated the regulation of its expression mediated by the PR/ER transcriptional complex. Also, we showed that NR2F1‐AS1 overactivation induced the quiescence‐like state in ER‐positive breast cancer cells. These findings bring favorable prospects for developing new predictive approaches and new therapeutic strategies.

## Conflict of interest

The authors declare no conflict of interest.

## **Author** **contributions**

ASC performed the experimental design and wrote the manuscript. ASC, TY, YK, and AM performed the methodology. ASC and YK involved in the investigation. ASC and YY analyzed the data. MO, HT, AS, KT, and FT involved in the clinical sample collection. FT, TO, and YY provided the funding acquisition. YY involved in the supervision.

## Supporting information


**Fig S1.** qPCR validation of other candidates of lncRNAs associated with recurrence.
**Fig S2.** A–C. Sequencing reads of TCGA_BRCA data for NR2F1‐AS1 in Luminal, HER2‐positive and TNBC subtypes accounting for recurrence cases (A), subtracted clinical cases with incidence of positive lymph nodes (B), age at initial diagnosis with 50 years as the delineation point (C).
**Fig S3.** Pearson correlation of ER, PR and ERBB2 versus NR2F1‐AS1 restricted to no recurrence samples.
**Fig S4.** Pearson correlation of ERα, ERβ, PR and ERBB2 versus NR2F1‐AS1 in 9 breast cancer cell lines.
**Fig S5.** Ki67 staining in NR2F1‐AS1‐transfected BT474 cells.
**Fig S6.** p21 and p27 levels in NR2F1‐AS1‐transfected BT474 cells.
**Fig S7.** GSEA analysis for BT474‐NR2F1‐AS1.
**Fig S8.** Analysis of NR2F1‐AS1 knockdown in MCF7 cells.
**Fig S9.** Phosphorylation levels of STAT1 and p38 MAPK in NR2F1‐AS1‐transfected BT474 cells.
**Fig S10.** Anoikis resistance of NR2F1‐AS1‐transfected BT474 cells.
**Fig S11.** Metastatic potential of NR2F1‐AS1.
**Fig S12.** Expression of NR2F1‐AS1 in a drug treatment with MCF7 cells after 5 days of combined administration of TAM and palbociclib (in a mol:mol ratio).

**Table S1.** Treatment information of 24 patients.
**Table S2.** List of primers and siRNAs.
