# Supplementary material for: Long non‐coding NR2F1‐AS1 is associated with tumor recurrence in estrogen receptor‐positive breast cancers
Source: Mol Oncol. 2020 Jun 8;14(9):2271–87. doi: 10.1002/1878-0261.12704 (PMC7463365; doi:10.1002/1878-0261.12704)
Supplement: Supplementary file 1 — Fig S1. qPCR validation of other candidates of lncRNAs associated with recurrence. Fig S2. A–C. Sequencing reads of TCGA_BRCA data for NR2F1‐AS1 in Luminal, HER2‐positive and TNBC subtypes accounting for recurrence cases (A), subtracted clinical cases with incidence of positive lymph nodes (B), age at initial diagnosis with 50 years as the delineation point (C). Fig S3. Pearson correlation of ER, PR and ERBB2 versus NR2F1‐AS1 restricted to no recurrence samples. Fig S4. Pearson correlation of ERα, ERβ, PR and ERBB2 versus NR2F1‐AS1 in 9 breast cancer cell lines. Fig S5. Ki67 staining in NR2F1‐AS1‐transfected BT474 cells. Fig S6. p21 and p27 levels in NR2F1‐AS1‐transfected BT474 cells. Fig S7. GSEA analysis for BT474‐NR2F1‐AS1. Fig S8. Analysis of NR2F1‐AS1 knockdown in MCF7 cells. Fig S9. Phosphorylation levels of STAT1 and p38 MAPK in NR2F1‐AS1‐transfected BT474 cells. Fig S10. Anoikis resistance of NR2F1‐AS1‐transfected BT474 cells. Fig S11. Metastatic potential of NR2F1‐AS1. Fig S12. Expression of NR2F1‐AS1 in a drug treatment with MCF7 cells after 5 days of combined administration of TAM and palbociclib (in a mol:mol ratio). [file MOL2-14-2271-s001.pdf]

Figure S1 Sanchez-Calle A et al.

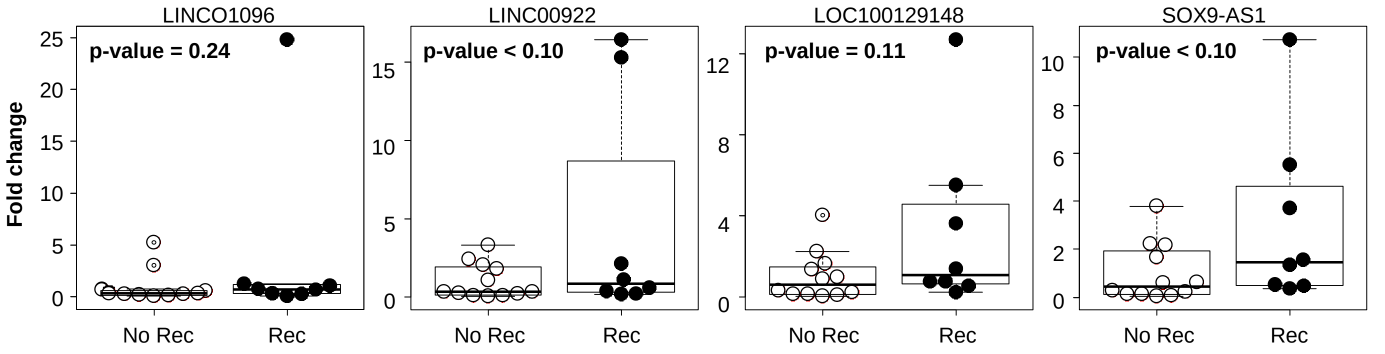

Figure S1 – qPCR validation of other candidates of lncRNAs associated with recurrence. Upper whisker: max, and lower whisker: min.

Figure S2 Sanchez-Calle A et al.

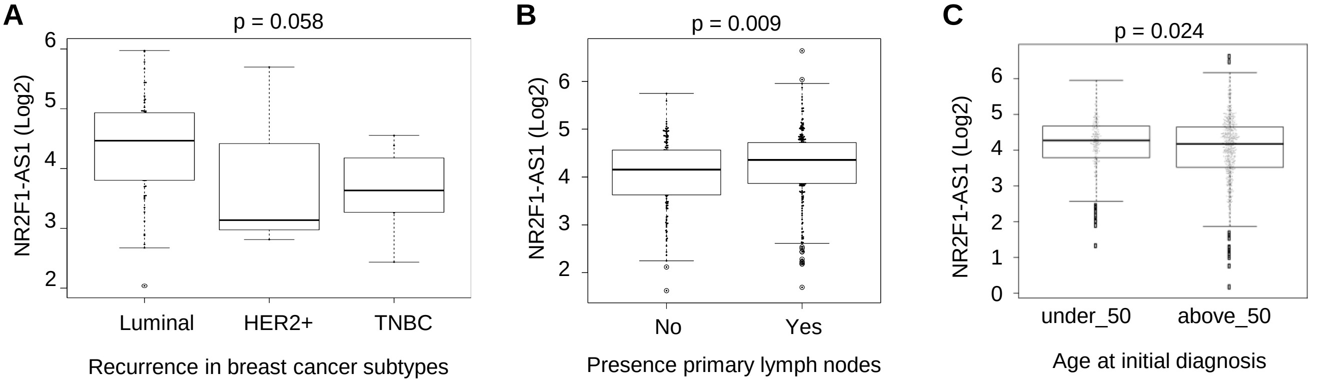

Figure S2 – A-C. Sequencing reads of TCGA_BRCA data for NR2F1-AS1 in Luminal, HER2-positive and TNBC subtypes accounting for recurrence cases (A), subtracted clinical cases with incidence of positive lymph nodes (B), age at initial diagnosis with 50 years as the delineation point (C). Upper whisker: max, and lower whisker: min.

Figure S3 Sanchez-Calle A et al.

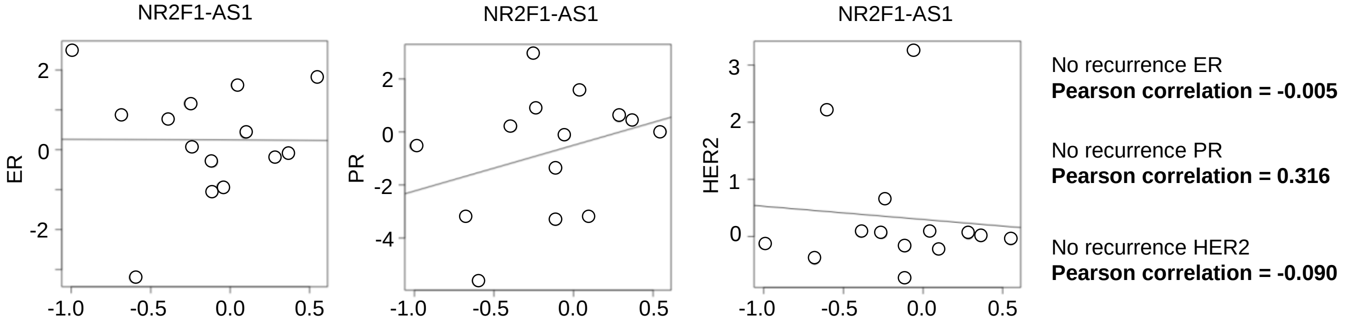

Figure S3 – Pearson correlation of ER, PR and ERBB2 versus NR2F1-AS1 restricted to no recurrence samples.

Figure S4 Sanchez-Calle A et al.

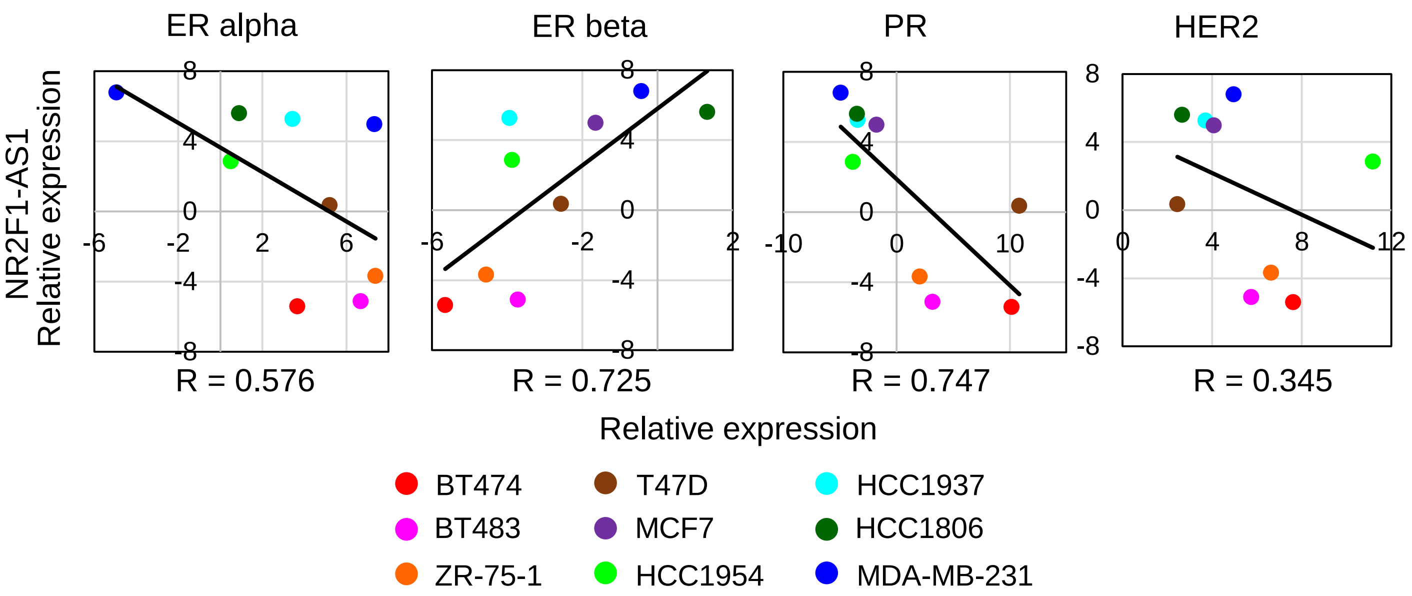

Figure S4 – Pearson correlation of ERα, ERβ, PR and ERBB2 versus NR2F1-AS1 in 9 breast cancer cell lines.

Figure S5 Sanchez-Calle A et al.

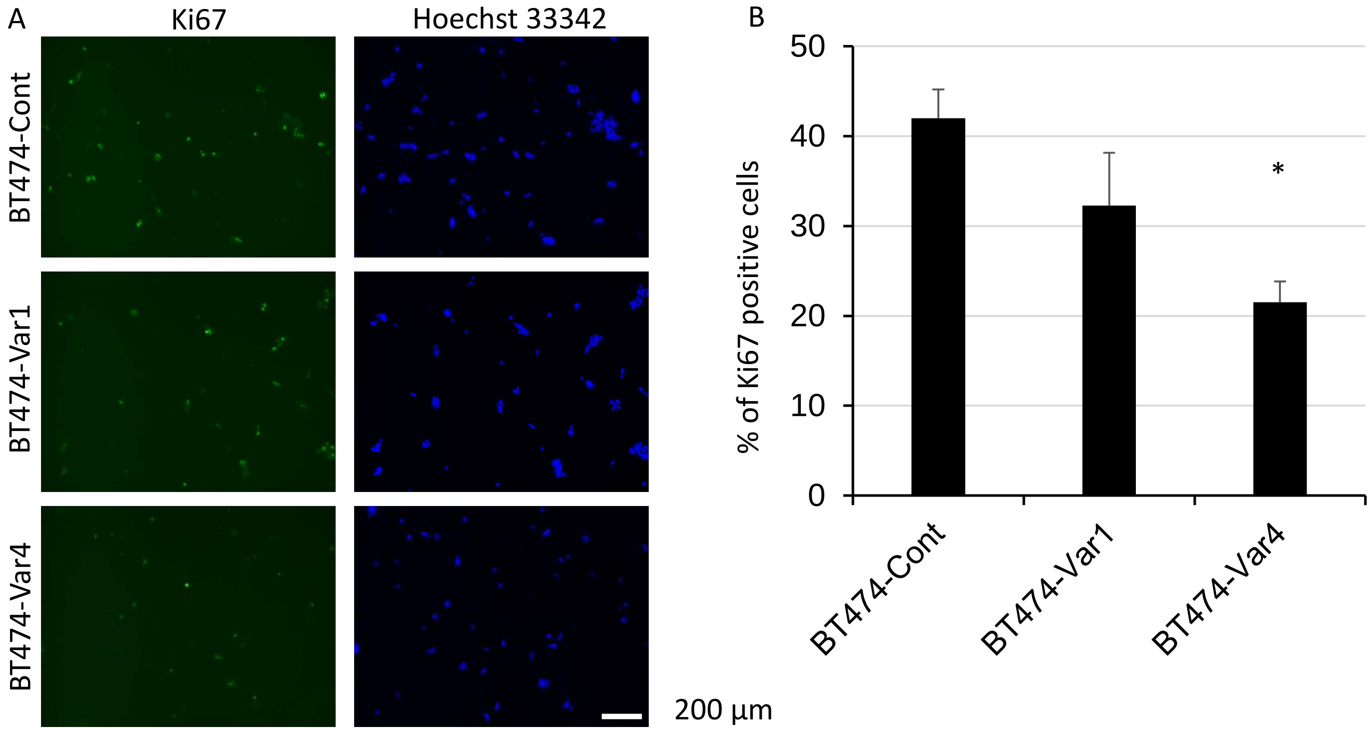

Figure S5 - Ki67 staining in NR2F1-AS1-transfected BT474 cells. A. Ki67 staining of BT474-control, BT474- NR2F1-AS1 Variant 1 and BT474- NR2F1-AS1 Variant 4. **B**. % of Ki67 positive cells in NR2F1-AS1-transfected BT474 cells. n = 3. Scale bar: 200 µm. Error bars: mean ± SD.

Figure S6 Sanchez-Calle A et al.

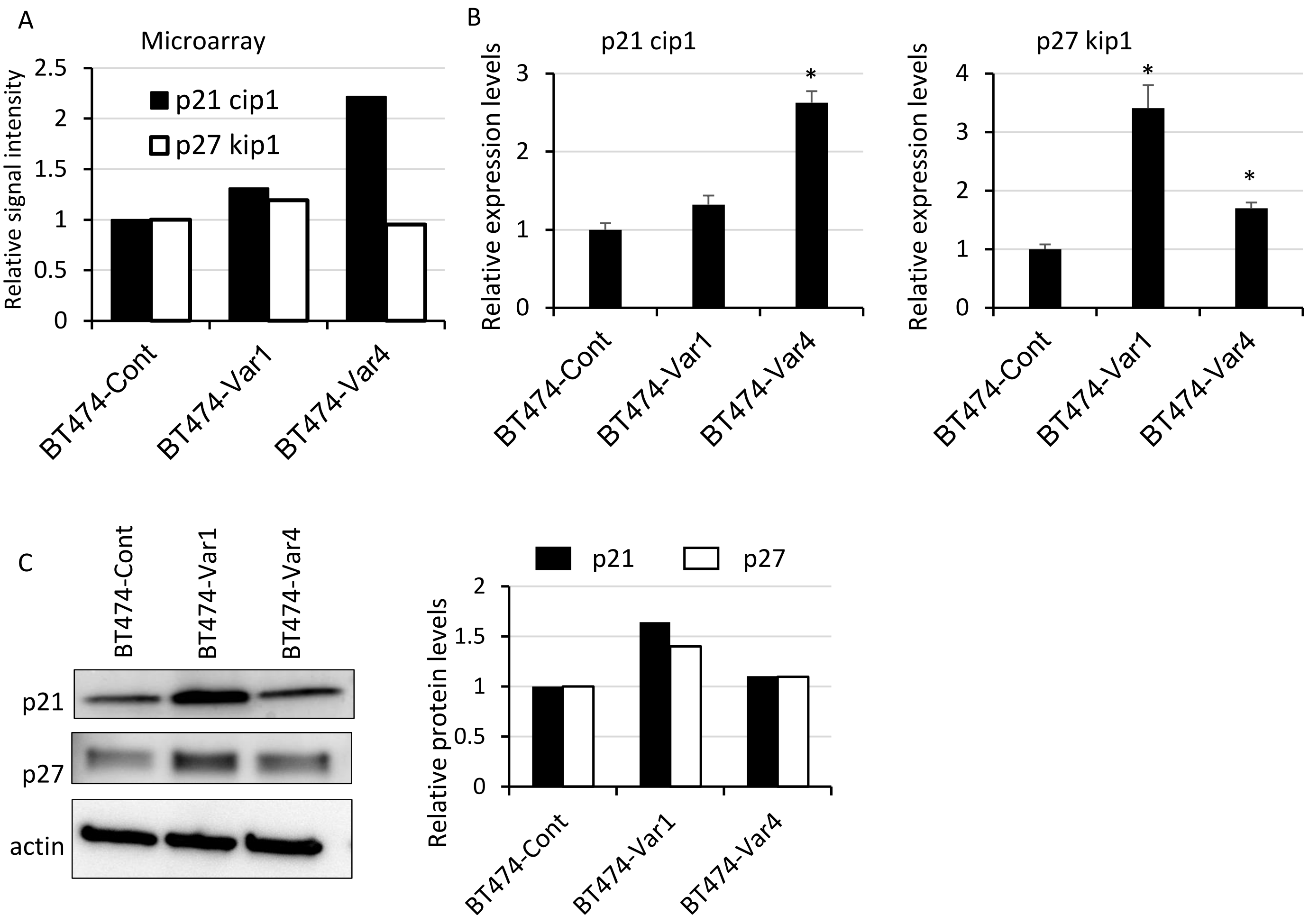

Figure S6 - p21 and p27 levels in NR2F1-AS1-transfected BT474 cells. A. relative signal intensity of p21 and p27 in microarray. The signal intensities of BT474-control set to 1.0. **B.** qRT-PCR analysis of p21 and p27 expression in NR2F1-AS1-transfected BT474 cells. *: $p < 0.05$ by Dunnett's test. **C.** western blotting of p21 and p27 protein levels in NR2F1-AS1-transfected BT474 cells. Right graph shows quantification of band intensity normalized with actin. Error bars: mean ± SD.

Figure S7 Sanchez-Calle A et al.

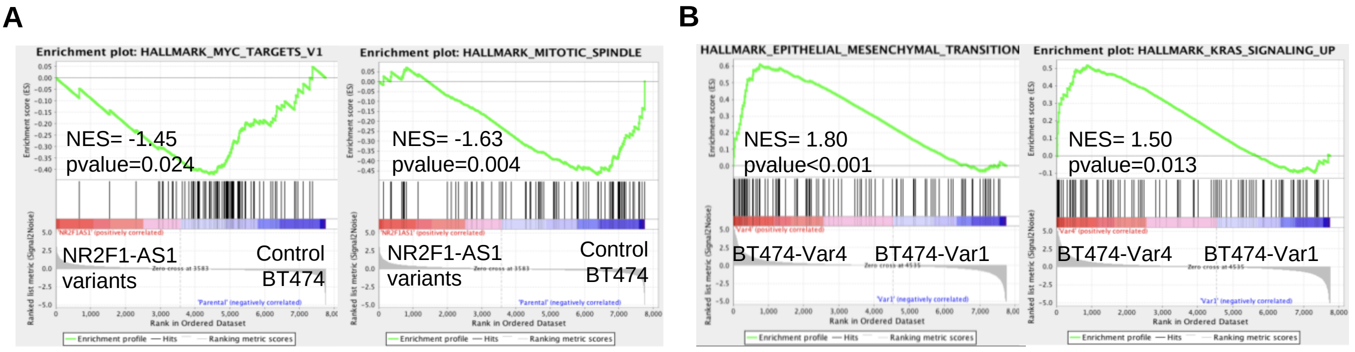

Figure S7 – GSEA analysis for BT474-NR2F1-AS1. A. Functional enrichment analysis by GSEA for BT474-Var1 and BT474-Var4 cells (in combination) versus Control BT474 cells ($p<0.05$); and **B.** functional enrichment analysis by GSEA for BT474-Var1 cells versus BT474-Var4 cells ($p<0.05$).

Figure S8 Sanchez-Calle A et al.

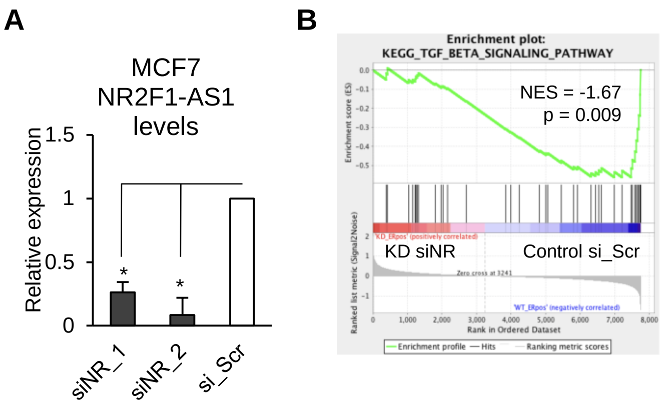

Figure S8 – Analysis of NR2F1-AS1 knockdown in MCF7 cells. A. Transient silencing of NR2F1-AS1 for 72 h with siRNA (siR_NR). Two different siRNA sequences were used in MCF7. Scramble siRNA (siR_Scr) is shown as a negative control. Error bars: mean ± SD. **B.** Functional enrichment analysis by GSEA for siR_NR versus control siR_Scr in MCF7 ($p<0.01$).

Figure S9 Sanchez-Calle A et al.

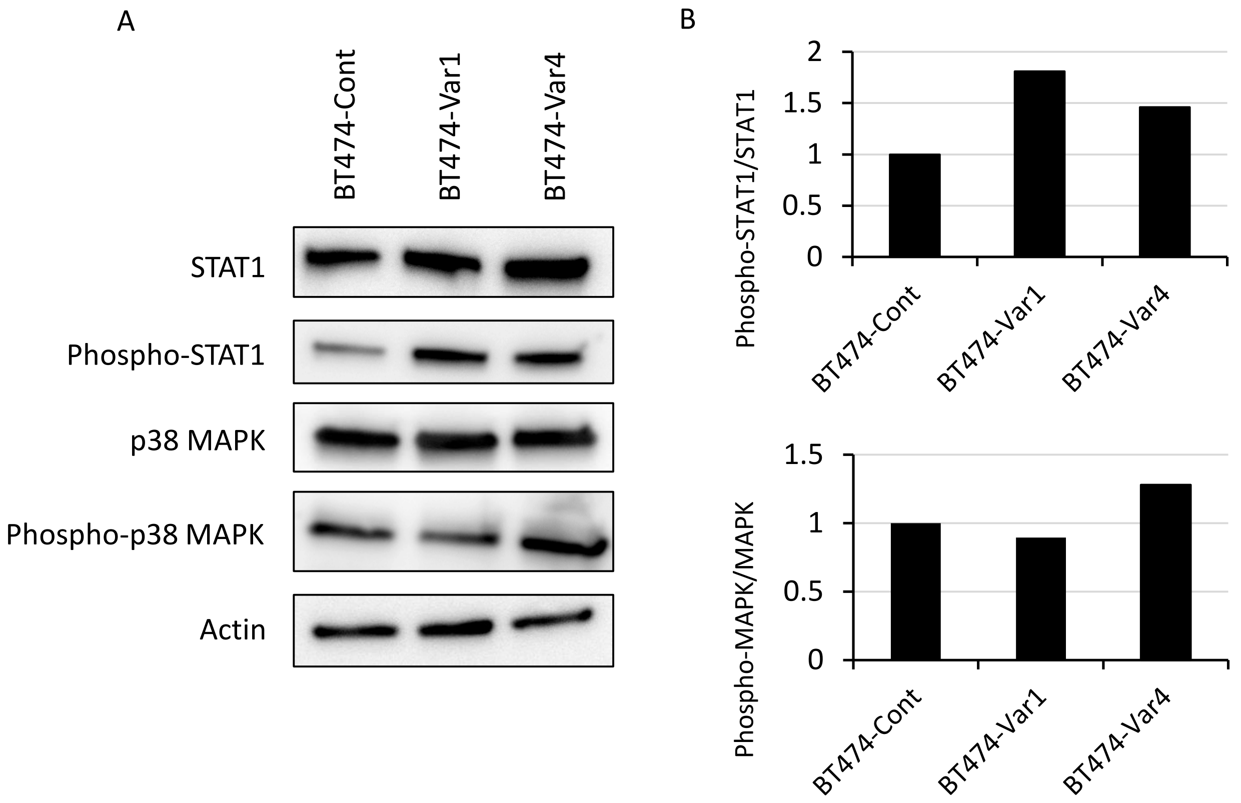

Figure S9 – Phosphorylation levels of STAT1 and p38 MAPK in NR2F1-AS1-transfected BT474 cells. A. western blot analysis of phospho-STAT1 and phosphor-p38 MAPK. Actin is loading control. **B.** Quantification of phosphorylated proteins. Phospho-STAT1 levels were normalized with STAT1, and phospho-p38 MAPK levels were normalized with p38 MAPK protein levels.

Figure S10 Sanchez-Calle A et al.

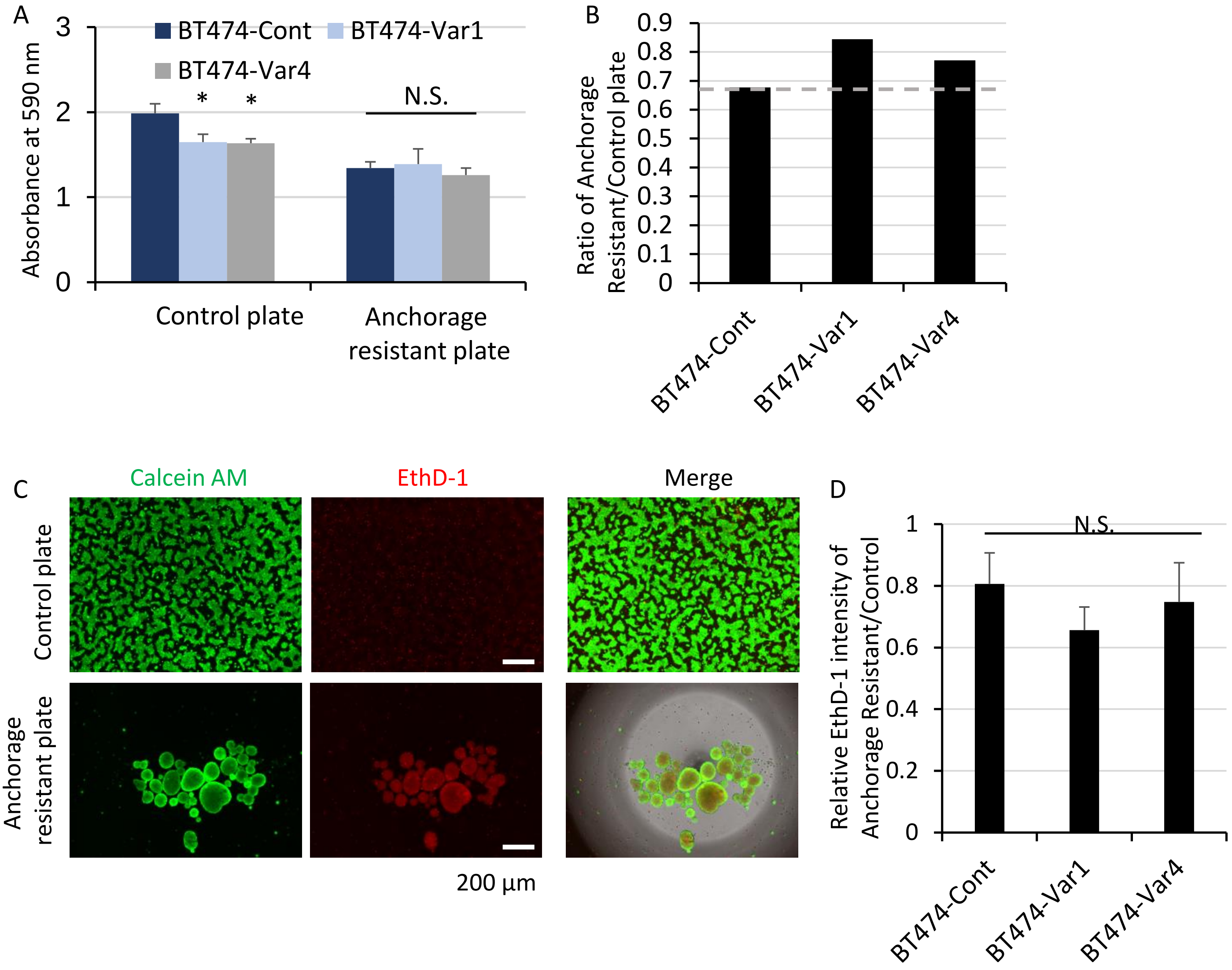

Figure S10 – Anoikis resistance of NR2F1-AS1-transfected BT474 cells. A. MTT assay of NR2F1-AS1-transfected BT474 cells in the normal and anchorage-resistant conditions. *: $p < 0.05$ by Dunnett's test. **B.** ratio of cell viability between normal and anchorage resistant to display anoikis resistance after NR2F1-AS1 transfection. **C.** Representative images of staining with Calcein AM (Detecting live cells) and Ethidium Homodimer (EthD-1, for the detection of cell death). **D.** Relative fluorescence levels of EthD-1 between normal and anchorage-resistant conditions, normalized with Calcein AM fluorescence. Error bars: mean ± SD.

Figure S11 Sanchez-Calle A et al.

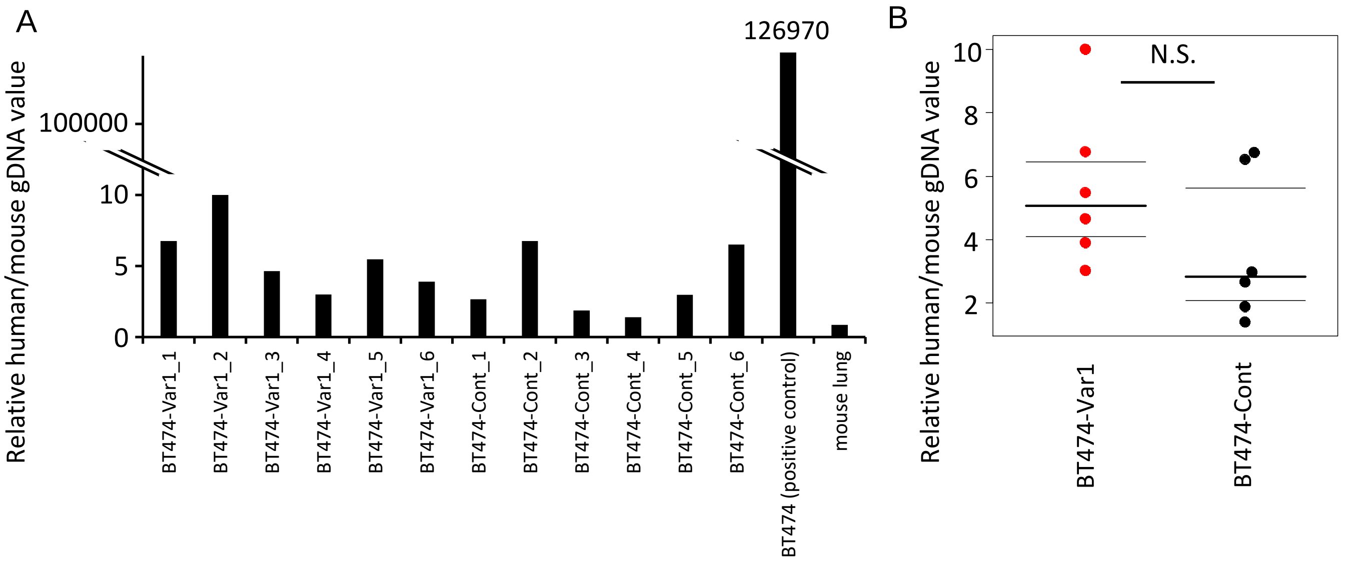

Figure S11 – Metastatic potential of NR2F1-AS1. A. NR2F1-AS1 Var1 or control plasmid-transfected BT474 cells were transplanted into immunodeficient mouse tail vein. Three days after injection, mouse lungs were collected, and gDNA was isolated. The number of human cells were estimated by qPCR comparing with human specific (Alu sequence) and mouse specific primers (mouse MHC I) as shown in Table S2 . Cultured BT474 cells: positive control and non-transplanted mouse lung: negative control (background signal). BT474-Var1: n = 6; BT474-Cont: n = 6. **B.** summary of estimated relative human cell rates in BT474-transplanted mouse lungs. this data also implicated the possibility of which NR2F1-AS1 positively functions to metastasize cancer cells. Bars represent 25th percentile, median and 75th percentile, respectively.

Figure S12 Sanchez-Calle A et al.

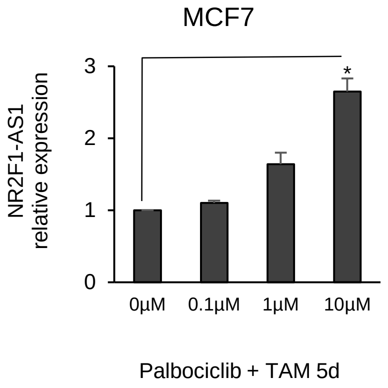

Figure S12 – Expression of NR2F1-AS1 in a drug treatment with MCF7 cells after 5 days of combined administration of TAM and palbociclib (in a mol:mol ratio). Error bars: mean ± SD.
