# Supplementary figures and images for: Long non‐coding NR2F1‐AS1 is associated with tumor recurrence in estrogen receptor‐positive breast cancers
Source: Mol Oncol. 2020 Jun 8;14(9):2271–87. doi: 10.1002/1878-0261.12704 (PMC7463365; doi:10.1002/1878-0261.12704)

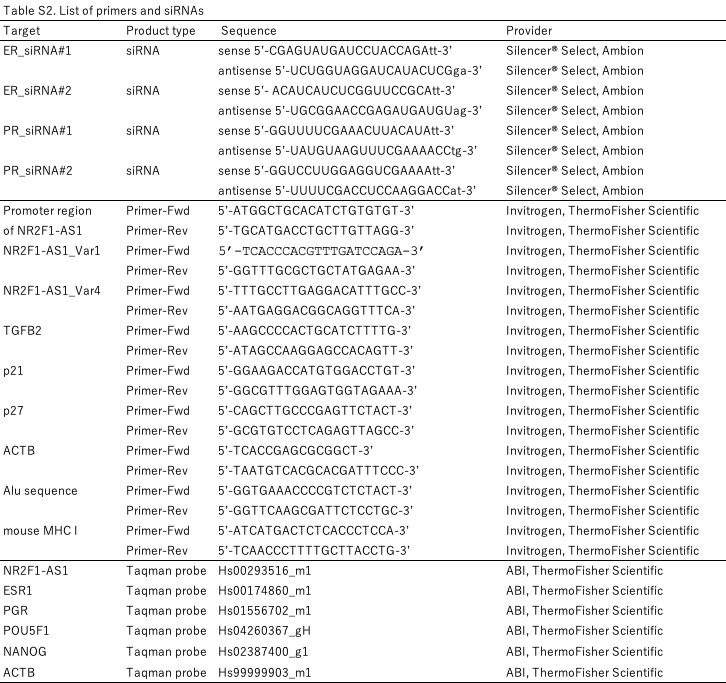

Supplement: Supplementary file 2 — Table S1. Treatment information of 24 patients. Table S2. List of primers and siRNAs. [file MOL2-14-2271-s002.docx]
